# Isolation and Characterization of Cold-Adapted PGPB and Their Effect on Plant Growth Promotion

**DOI:** 10.4014/jmb.2105.05012

**Published:** 2021-07-15

**Authors:** Mingyuan Li, Jilian Wang, Tuo Yao, Zhenlong Wang, Huirong Zhang, Changning Li

**Affiliations:** 1 College of Grassland Science, Gansu Agricultural University, Lanzhou 730000, P.R. China; 2 College of Biologic and Geographic Sciences, Kashi University, Kashi 844000, P.R. China; 3 Key Laboratory of Ecology and Biological Resources in Yarkand Oasis of Education of Xinjiang Uygur Autonomous Region, Kashi 844000, P.R. China

**Keywords:** Plant growth-promoting bacteria (PGPB), screen, cold-adapted, the Qilian Mountains, alpine grasslands

## Abstract

Cold-adapted plant growth-promoting bacteria (PGPB) with multiple functions are an important resource for microbial fertilizers with low-temperature application. In this study, culturable cold-adapted PGPB strains with nitrogen fixation and phosphorus solubilization abilities were isolated. They were screened from root and rhizosphere of four dominant grass species in nondegraded alpine grasslands of the Qilian Mountains, China. Their other growth-promoting characteristics, including secretion of indole-3-acetic acid (IAA), production of siderophores and ACC deaminase, and antifungal activity, were further studied by qualitative and quantitative methods. In addition, whether the PGPB strains could still exert plant growth-promoting activity at 4°C was verified. The results showed that 67 isolates could maintain one or more growth-promoting traits at 4°C, and these isolates were defined as cold-adapted PGPB. They were divided into 8 genera by 16S rRNA gene sequencing and phylogenetic analysis, of which *Pseudomonas* (64.2%) and *Serratia* (13.4%) were the common dominant genera, and a few specific genera varied among the plant species. A test-tube culture showed that inoculation of *Elymus nutans* seedlings with cold-adapted PGPB possessing different functional characteristics had a significant growth-promoting effect under controlled low-temperature conditions, including the development of the roots and aboveground parts. Pearson correlation analysis revealed that different growth-promoting characteristics made different contributions to the development of the roots and aboveground parts. These cold-adapted PGPB can be used as excellent strain resources suitable for the near-natural restoration of degraded alpine grasslands or agriculture stock production in cold areas.

## Introduction

Grasslands are multifunctional ecosystems covering the largest land area with the most extensive distribution on Earth. They play an irreplaceable role in the balance of terrestrial ecosystems, soil and water conservation, and biodiversity maintenance. China has the second-largest grassland area in the world. Since the mid-20th century, due to the combined impacts of human activities and climate change, most grassland has been degraded to varying degrees, leading to a series of problems, such as grassland habitat degradation and biodiversity imbalance, which seriously threaten the sustainable development of animal husbandry and ecology [1, 2]. With the degeneration of grasslands, the physicochemical properties, enzyme activities, and microbial community structure of soil are significantly changed [3, 4]. Studies have shown that the diversity and complexity of soil microbial communities are closely related to grassland ecosystem function and nutrient cycling. In contrast, the extinction of complex ecological associations among soil microbial communities in grasslands can impair ecosystem functioning [5]. Although chemical fertilization can improve grassland productivity, its long-term and excessive use leads to serious environmental problems, such as accelerated deterioration of soil properties and imbalances in nutrient proportions [6].

Plant growth-promoting bacteria (PGPB) are a group of beneficial microorganisms that widely exist in the root or rhizosphere soil of plants and are closely related to the metabolism of the host plant roots. They carry out plant growth-promoting activities such as fixing nitrogen, solubilizing phosphate, and producing antibiotics and plant growth hormones [7, 8]. In different model systems, PGPB promote plant growth through direct nutrient regulation [9, 10] and show antagonism against harmful pathogenic bacteria through a complex rhizosphere interaction network [11]. Additionally, they induce plants to enhance their resistance to biological or abiotic stress [12-14] and are of great significance in the biogeochemical cycling of elements and soil remediation [15, 16]. Excellent strains are the basis of microbial fertilizers and many such strains with multiple functions and high plant growth-promoting activity have been proven to be effective in producing biofertilizers that can replace chemical fertilizers [9, 17, 18]. Although there have been many commercial microbial fertilizers developed, different plant species possess specific PGPB community structures due to their different habitats and planting areas, so microbial fertilizers are not universal. In addition, biological fertilizers composed of a single functional strain are often ineffective and have low market recognition, preventing them from meeting the development needs of modern agriculture stock [19]. Therefore, the isolation and identification of PGPB resources with multiple functions and high plant growth-promoting activity from different habitats still represent a bottleneck in the development of efficient microbial fertilizers and new pesticides.

Temperature is a key factor affecting plant growth. Low temperature stresses plants and affects the growth and activity of microorganisms. Studies have shown that the inoculation of cold-adapted PGPB microbial inoculants significantly promoted plant growth under low-temperature conditions [20, 21]. Both agricultural cultivation in high latitude cold areas and off-season greenhouse vegetables require biofertilizers that can function at low temperatures. Therefore, the screening of cold-resistant PGPB is of great significance. The Qinghai-Tibet Plateau is the main distribution area of alpine grassland in China, accounting for approximately 1/4 of China's land area and serving as a barrier to China's water resources and ecological security. The Qilian Mountain range is located on the northeastern border of the Qinghai-Tibet Plateau, whose unique geographical environment and climate conditions harbor unique biological resources and biological phenomena. The main objectives of this study were to (1) select cold-adapted PGPB from four dominant grass species in the alpine grasslands of the Qilian Mountains with various functions such as nitrogen fixation, phosphorus solubilization, auxin secretion, ACC deaminase production and biological control agents; (2) identify the genetic diversity of cold-adapted PGPB from different plant species; and (3) verify the effects of inoculation with PGPB on *Elymus nutans* by test-tube culture. In addition, the results herein should provide microbial resources and basic data to assist in developing multifunctional microbial inoculants with high plant growth-promoting activity at low temperatures and potential applications in cold areas.

## Materials and Methods

### Study Sites and Sampling

The field investigation was conducted at two sites (Table 1), including site TZ, the Tianzhu alpine meadow ecosystem experimental station of Gansu Agricultural University, Tianzhu Tibetan Autonomous County, Gansu Province, China, and site MY, the Haibei alpine meadow ecosystem research station of the Chinese Academy of Sciences, Menyuan County, Qinghai Province. Site TZ is located at the eastern end of the Qilian Mountains, ≥ 0°C annual average cumulative temperature is 1380°C, and the annual evaporation is approximately 4 times the precipitation. Site MY is located in the Qilian Mountain valley, which is cold and dry in winter and cool and humid in summer.

Material samples of four dominant and subdominant forages, *Bromus inermis* Leyss (B), *E. nutans* (E), oat (*Avena sativa* L.) (G), and *Poa annua* L.(J), were collected at the two sites. A multipoint sampling method was used that included an "S-type" route to randomly select 12 sites in the sample region with a distance of not less than 20 m between two sites. Three healthy individual plants (with vigorous growth and a consistent canopy size) were selected at each site. The roots were dug deeply and gently shaken to remove the bulk soil while retaining the tightly bound soil (1-3 mm). The roots were cut out and placed into sterile containers. The same plant rhizosphere material collected from every 3 adjacent sites was mixed into one sample, resulting in 4 replicates of each plant species. A total of 16 samples were immediately transported in a cooling box to the laboratory.

### Screening of Cold-Adapted PGPB

**Screening of plant growth-promoting rhizobacteria.** A root sample of about 10 g was suspended in a triangle flask containing 90 ml sterile saline with sterile glass beads (φ3 mm) and agitated on a rotary shaker (180 rpm) for 60 min at 15°C. One milliliter of this soil suspension was used to prepare serial 10-fold dilutions with sterile saline. A 200 μl aliquot of 10^-5^, 10^-6^, 10^-7^, and 10^-8^ dilutions was used for plating in triplicate on nitrogen-deficient malate (NFM) agar [22] to isolate nitrogen-fixing bacteria. The National Botanical Research Institute’s phosphate (NBRIP) agar [23] and Mongina organic culture medium [22] were used for the screening of strains dissolving inorganic and organic phosphorus. The plates were incubated for 5-7 days at 15°C to observe the colony growth and phosphate-dissolving rings. Pure cultures were obtained by serial subculturing.

All isolates were cultured at 4°C, from which the strains with fast growth rates that were still positive for various functions were defined as cold-adapted PGPB.

**Screening of plant growth-promoting endophytic bacteria.** The roots samples were washed three times with distilled water to remove impurities, and the damaged portions were sealed with paraffin wax. The root surface was treated with 0.1% (w/v) HgCl_2_ for 5 min. The paraffin-coated root segments were cut off under aseptic conditions. A root sample of about 1 g was ground with a sterilized mortar and pestle, suspended in a 15-ml sterile centrifuge tube containing 9 ml sterile saline, and agitated on a rotary shaker for 1 min at room temperature. The suspension was diluted with sterile saline solution to 10-6 to isolate nitrogen-fixing and phosphate-solubilizing strains according to the method used with rhizobacteria.

### Determination of Plant Growth Promotion Characteristics of PGPB Strains

**Nitrogen fixation ability.** The nitrogenase activity of the bacteria isolated from the NFM agar medium was measured by the acetylene reduction method [24]. The bacterial cells were centrifuged and collected, and then the protein content was determined by the Bradford method [25]. To test the multiple functions of isolates, all phosphate-solubilizing strains were inoculated onto NFM plates to verify the nitrogen fixation ability, and the positive strains were quantitatively determined by the same method as above.

**Phosphate solubilization ability.** The isolates capable of dissolving inorganic and organic phosphorus were incubated in liquid NBRIP medium and Mongina medium [23] at 15°C with shaking (180 rpm) for 7 days. The pH and soluble phosphorus content in the culture solution were then measured according to the molybdenum blue colorimetric method [26]. Moreover, the nitrogen-fixing strains were inoculated onto NBRIP and Mongina plates to verify their ability to dissolve phosphorus, and the positive strains were then quantitatively determined by the same method as above.

**IAA production ability.** Indole-3-acetic acid (IAA) production in all isolates was detected through qualitative [27] and quantitative tests. The strains were inoculated into King's liquid media [28] and incubated at 15°C with shaking (180 rpm) for 3 days. Approximately 50 μl of the supernatant was mixed with 50 μl of Salkowski’s reagent. After 30 min, the color change from yellow to pink was considered positive. Quantitative measurement of IAA was conducted by high-performance liquid chromatography [29].

### ACC Deaminase Activity Detection

All isolates were inoculated on ADF and DF medium plates [30] and cultured at 15°C for 7 days. The growth of the ADF medium was significantly better than that in the DF medium, indicating that they could produce 1-aminocyclopropane-1-carboxylic acid (ACC) deaminase and grow with ACC as the sole nitrogen source. According to the method previously described [30] with slight modifications, ACC deaminase activity was determined. The protein concentration was determined by the method of Bradford [25]. Enzyme activity (μmol/mg•protein•h) referred to the number of μmol of a-ketobutyrate produced when the enzyme ACC deaminase cleaves ACC in unit time.

### Siderophore Detection

The ability of all isolates to produce siderophores was evaluated through both qualitative and quantitative methods. All isolates were cultured on MSA-CAS plates (MSA medium containing chrome azurol S (CAS) shuttle solution) [31] and incubated at 15°C for 3-5 days. After incubation, the appearance of a yellow/orange halo around the bacterial colonies was considered positive.

Siderophore concentrations were determined by measuring the OD at 630 nm. The bacterial cells were cultured in liquid medium for iron-deficient MSA and incubated at 15°C with shaking (180 rpm) for 48 h. The cultures were then centrifuged at 13,000 r/min for 10 min, 2 ml of the supernatant was mixed with the blue dye CAS azurol S (CAS) reagent in the same volume, and the absorbance of all samples at 630 nm (As) was determined after standing at room temperature for 1 h. The control group (Ar) was the absorbance of reference (CAS reagent). The siderophore quantities were measured as % of siderophore units by the formula: % of siderophore units (SU) = [(Ar-As)/Ar] *100%.

### Antifungal Activity

The inhibitory effect of siderophore-producing bacteria against four plant pathogens (Fusarium oxysporum, Rhizoctonia solani, Helminthosporium tritici-vulgaris and Alternaria solani) was measured by the plate confrontation method [9]. There were 3 repeats of the treatment, and the control group was plates inoculated only with a pathogen. The inhibition rate was calculated by the following formula: inhibition rate (%) = (D−d)/D × 100%. 'D'and 'd' show the colony diameters of plant pathogens in the control and treatment groups, respectively.

### Identification and Phylogenetic Analysis of Cold-Adapted PGPB Strains

A DNA extraction kit (TaKaRa, Japan) was used to extract genomic DNA from the cold-adapted PGPB strains. The 16S rRNA genes were amplified from genomic DNA by PCR using the universal primers 27F and 1492R [32]. The PCR products were checked by 1% agarose gel electrophoresis and were subsequently sequenced using the dideoxy chain-termination method at Sangon Biotech Co., Ltd. (China). The sequencing results were verified with the EzBioCloud database (http://www.ezbiocloud.net). Furthermore, the phylogenetic tree was constructed using the neighbor-joining method implemented in the MEGA 7.0 software package, and the topology of the tree was evaluated by a bootstrap test with 1000 replicates [33].

### Inoculation Experiment

The tested strains were 12 cold-adapted PGPB with potential plant growth-promoting characteristics and different classification statuses, including TZnJn3 and TZnJn4 with the highest nitrogenase activity, MYmG3 and MYmG4 with the highest organic phosphate solubilization ability, MYpJn1 and MYpJn8 with the highest inorganic phosphate solubilization ability, TZnB16 and TZmJ2 with the highest IAA production ability, MYnB4 and MYnE2 with the highest siderophore production ability, and TZnG2 and MYpJn8 with the highest ACC deaminase activity. Before planting, the test strains were separately inoculated into LB liquid medium and incubated at 15°C with shaking (180 rpm) for 24-36 h. The bacterial suspension was then diluted with sterilized LB liquid medium to achieve an OD_600_ of 0.8 (viable cell count remained at 10^10^ CFU/ml), which was the final volume ratio used as the inoculum.

Full and uniform-sized seeds of *E. nutans* were selected, sterilized with 1% NaClO for 5 min, rinsed three times with sterilized water, and then incubated at 25°C on a water agar plate. When the bud length was 2-3 cm, the seedlings were then transplanted to a long glass tube (4 × 45 cm) containing 70 ml Hoagland nutrient solution (made semisolid with 0.2% agar to simulate soil breathability). For phosphate-dissolving strains, the phosphorus in the culture medium was replaced by insoluble tricalcium phosphate and soybean lecithin. After 2-3 days of growth, the seedlings were inoculated with 1% (v/v) PGPB inoculum and inactivated PGPB inoculum, respectively. Seedlings were inoculated with an equal volume of sterile LB medium as a control, and there were 5 repeats of the treatment and control groups. According to the average temperature data of grass returning to green stage in the sampling area, the seedlings were placed in random order in an artificial climate incubator (Ningbo Jiangnan Instrument Factory, China) at 15°C with light (14 h)/4°C with dark (10 h) cycles (light intensity at 4,000 lx) and a humidity of 60-70%. Sterile water was replenished regularly. After 28 days of coculture with bacterial inoculants, we measured the plant indexes to verify the growth-promoting effect, including the roots (root length, root average diameter, root surface area, root dry weight, number of root tips) and aboveground parts (stem height, stem diameter, stem dry weight).

### Statistical Analysis

SPSS version 21.0 and Origin 2019 software were used for statistical analysis and drawing of experimental data. Data were analyzed using analysis of variance (ANOVA) and Duncan's new multiple range test. Pearson correlation analysis was used to analyze the relationship between plant growth-promoting characteristics and the promoting effect of PGPB inoculants. Data are presented as the means ± standard deviation.

## Results

### Screening of Cold-Adapted PGPB Strains

A total of 285 PGPB strains with nitrogen fixation and phosphorus solubilization capacities were screened from the root and rhizosphere samples. Among them, there were 153, 71, and 61 strains of nitrogen-fixing, inorganic and organic phosphorus-dissolving bacteria, respectively (Table 2). Except for *E. nutans*, the number of nitrogen-fixing strains in the rhizosphere was greater than that in the roots. In all plant samples, the number of phosphate-dissolving bacteria isolated from the rhizosphere was greater than that from the root. All isolates were cross-inoculated to qualitatively analyze all the growth-promoting characteristics. The positive strains were cultured at 4°C, after which the strains with fast growth rates that were still positive for various functions were selected. As a result, 67 strains that could grow at 4°C and had plant growth-promoting traits were identified and defined as cold-adapted PGPB.

### Plant Growth-Promoting Characteristics

**Fig. 1:**
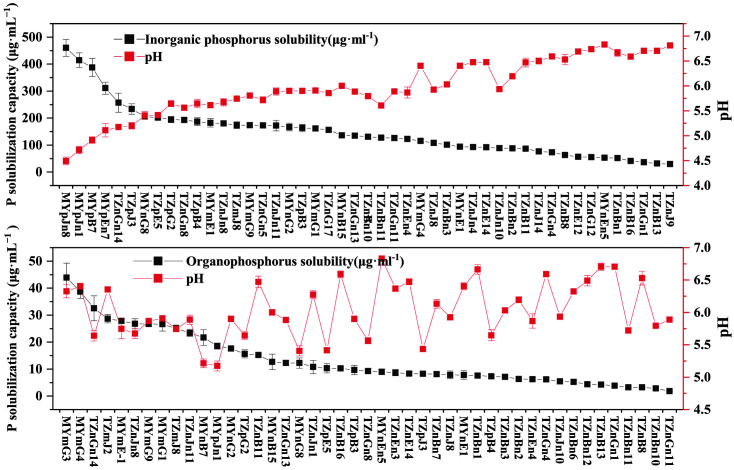
Relationship between phosphorus solubility and pH.

Sixty-four out of sixty-seven cold-adapted PGPB strains had nitrogen fixation ability. The nitrogenase activity was between 110.85-907.31 nmol/mg•protein•h, and strain TZnJn4 had the highest activity (Table 3). The organic phosphate solubility of forty-five strains ranged from 1.81 to 43.89 μg/ml, with a medium pH ranging from 5.17-6.83, and MYmG3 had the highest organic phosphate solubility. The inorganic phosphate solubility of forty-seven PGPB ranged from 29.84 to 31.34 μg/ml, with a medium pH ranging from 4.49-6.85, and MYpJn8 had the highest inorganic phosphate solubility. The inorganic phosphate solubility was negatively correlated with pH. The lower the pH was, the greater the inorganic phosphate solubility, but there was no linear relationship (Fig. 1). Thirty-two strains could secrete IAA, with contents between 3.24-48.95 μg/ml, and TZnB16 had the highest capacity. The siderophore concentrations of thirty-five strains were between 0.037-0.739 μg/ml. The higher the siderophore concentration was, the lower the SU values, and MYnB4 showed the highest siderophore production. Five strains had ACC deaminase activity, and the enzyme activity ranged from 1.21 to 3.24 μmol/(mg•protein•h).

The inhibition abilities of strains producing siderophores against different plant pathogens are shown in Table 3. It was found that thirty strains effectively inhibited *F. oxysporum*, and the inhibition rates ranged from 11.33% to 55.33%. Among them, strain MYnB15 had the highest inhibition rate. The numbers of PGPB strains that effectively inhibited *R. solani*, *H. tritici-vulgaris*, and *A. solani* were 28, 21, and 24, respectively, with inhibition rates of 17.19-56.52%, 6.22-54.44%, and 23.41-56.52%, respectively. Among them, strains MYnB4 and TZnE14, MYnB4, and MYnB15 had the highest inhibition ability. In general, among all cold-adapted PGPB strains, 63 strains had more than two growth-promoting functions, accounting for 94.03%, 56 strains had more than three functions, accounting for 83.58%, 35 strains had more than four functions, accounting for 52.24%, and 7 strains had more than six functions, accounting for 10.45%. PGPB strains with multifunctional characteristics were more suitable for the development of broad-spectrum microbial fertilizers.

### Identification and Phylogenetic Analysis of Cold-Adapted PGPB Strains

**Fig. 2:**
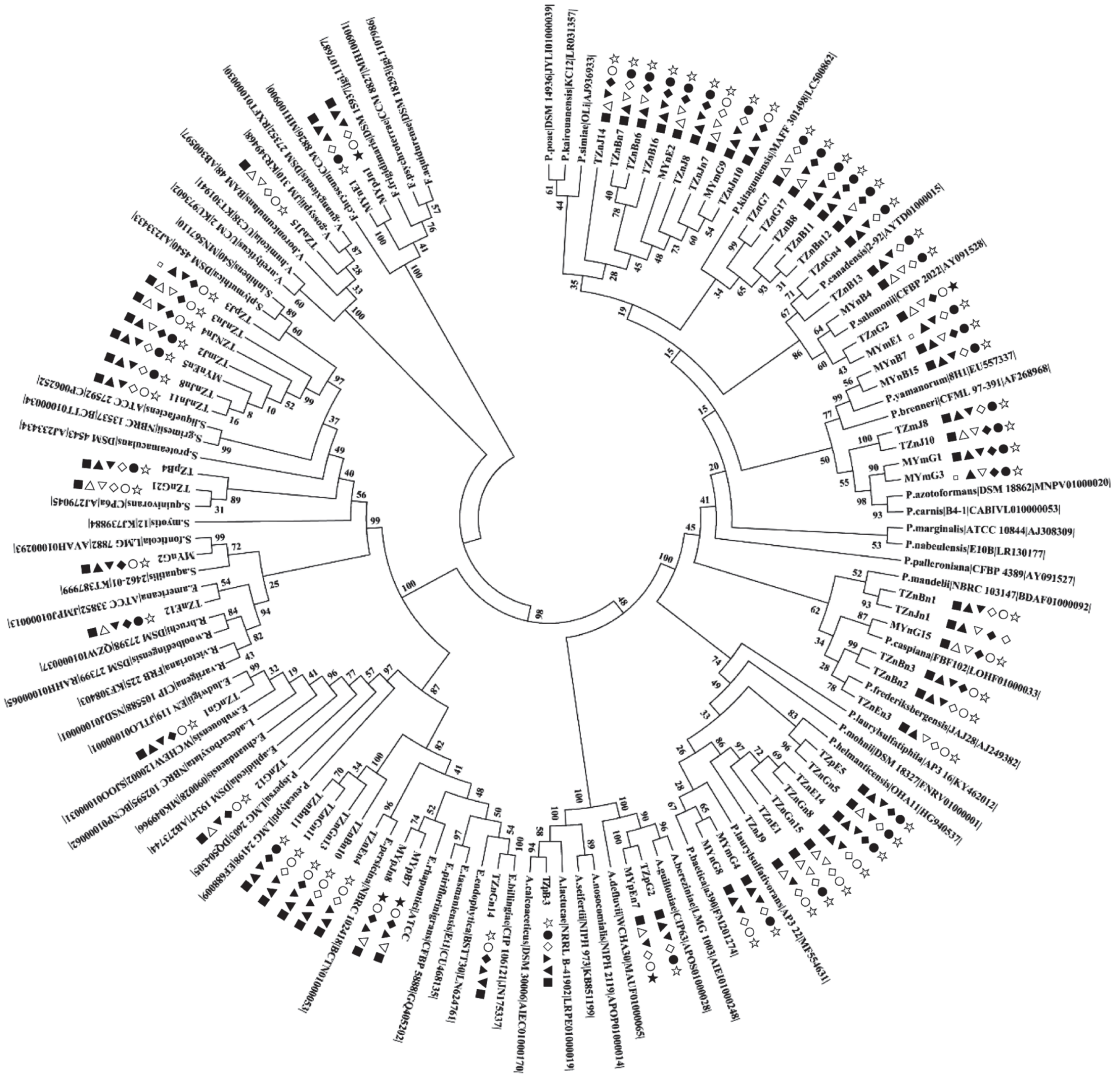
Phylogenetic analysis of 16S rRNA gene sequences of the cold-adapted PGPB strains. The following symbols indicated the potential plant growth-promoting traits of isolates: ■nitrogen fixation, □no nitrogen fixation, ▲organophosphorus solubilization, △no organophosphorus solubilization, ▼inorganic phosphorus solubilization, ▽no inorganic phosphorus solubilization, ◆IAA production, ◇no IAA production, ●siderophore production, ○no siderophore production, ★ACC deaminase production, ☆no ACC deaminase production.

**Fig. 3:**
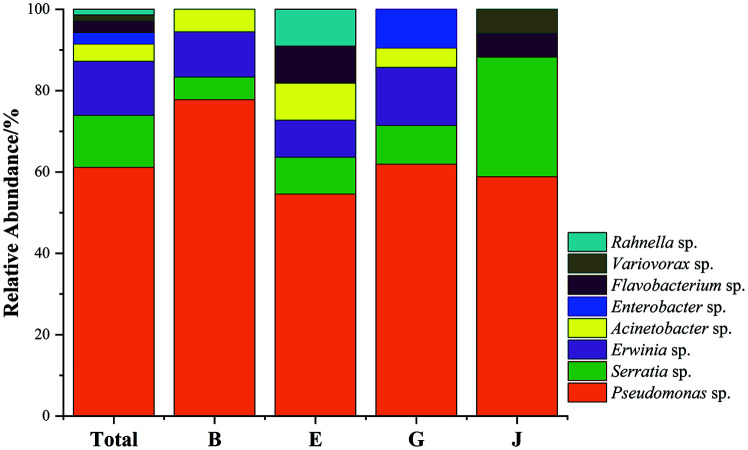
The bacterial community structures of the cold-adapted PGPB at the genus level. Total: Isolates from four grasses species, B: Isolates from *Bromus inermis* Leyss, E: Isolates from *Elymus nutans*, G: Isolates from *Avena sativa* L., J: Isolates from *Poa annua* L.

Thirty-eight of the 67 cold-adapted PGPB strains were from the rhizosphere, and 29 strains were from the root. The 16S rRNA gene sequences of the cold-adapted PGPB were analyzed, and phylogenetic trees were constructed (Fig. 2). All strains were divided into 8 genera, of which *Pseudomonas* was the dominant genus, accounting for 64.2%, and *Serratia* was the subdominant genus, accounting for 13.4%. However, the abundances of *Erwinia*, *Acinetobacter*, *Enterobacter*, *Flavobacterium*, *Variovorax*, and *Rahnellad* were low in the rhizosphere soil of the four plants, varying among plant species (Fig. 3). Specifically, *Erwinia* and *Acinetobacter* were not detected in the rhizosphere of *P. annua* L. Two *Enterobacter* strains were isolated only from the root and rhizosphere soil of *A. sativa* L. Two *Flavobacterium* strains were isolated only from the rhizosphere of *E. nutans* and the root of *P. annua* L, respectively. Two strains, *Variovorax* and Rahnella, were only isolated from the rhizosphere of *E. nutans*, and *P. annua* L. *Pseudomonas* was the common dominant genus of the samples, accounting for 54.5-77.8% of the different plant species.

### Inoculation Experiment

**Fig. 4:**
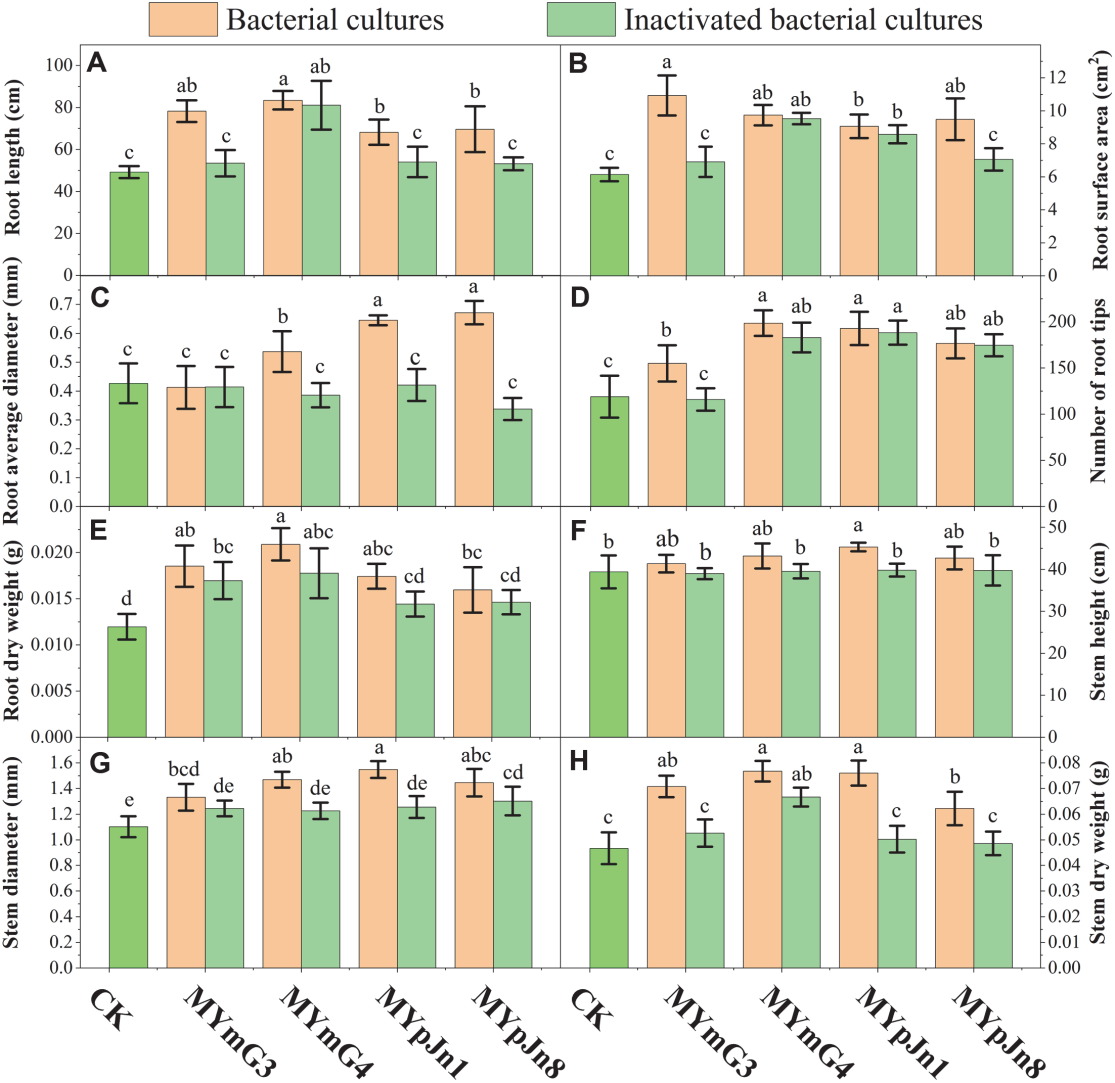
Effects of phosphate-dissolving strains on the growth of plants. Bacterial cultures: inoculation with strain cultures of MYmG3, MYmG4, MYpJn1, and MYpJn8. Inactivated bacterial cultures: inoculation with inactivated strain cultures of MYmG3, MYmG4, MYpJn1, and MYpJn8. CK: inoculation with sterilized LB medium.

Compared with the control group, all phosphate-solubilizing bacteria significantly increased the RL and RSA by 8.08%-69.47% and 12.52%-77.89%, respectively (Figs. 4A and 4B; Table S1). Furthermore, inoculation with non-inactivated strain cultures was more effective than that with inactivated strains. Inoculation with MYmG4 and MYmG3 had the most significant effect on the RL and RSA, which increased by 69.47% and 77.89%, respectively. Inoculation with phosphate-solubilizing bacteria had different effects on RAD and NRT. Except for strain MYmG3, the effects of MYmG4, MYpJn1, and MYpJn8 were positive for RAD, which increased by 25.76%, 51.29%, and 57.38%, respectively (Figs. 4C and 4D; Table S1). However, inoculation with inactivated strains all showed weak inhibition for RAD, among which MYpJn8 exhibited the highest inhibition, up to 20.84%. Except for inoculation with MYmG3, other treatment groups significantly increased the NRT by 30.25-66.95%.

Except for inactivated strain MYmG3, inoculation with other strains and inactivated strains increased the SH, SD, SDW, and RDW (Figs. 4E-4H; Table S2) by 0.43-15.05%, 11.82-40.91%, 4.26-63.83%, and 16.67-75.00%, respectively. Among them, strain MYpJn1 had the strongest effect on SH and SD, and MYmG4 exhibited the highest capacity for SDW and RDW. In contrast, the growth-promoting effect of non-inactivated strains was significant compared with that of inactivated strains (*p* < 0.05).

**Fig. 5:**
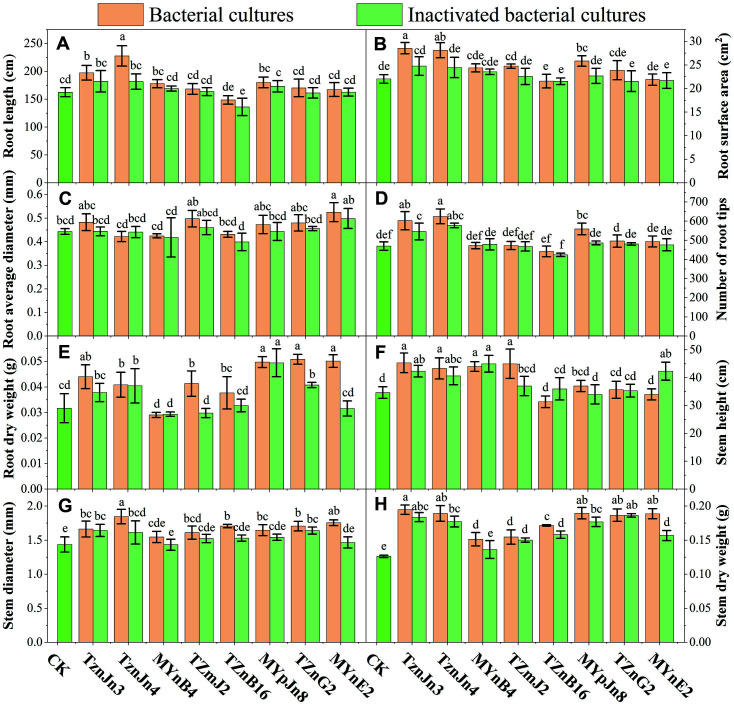
Effects of inoculation with nitrogen-fixing, IAA-, siderophore- and ACC deaminase-producing strains on growth characteristics of plants. Bacterial cultures: inoculate with strain cultures of TZnJn3, TZnJn4, MYnB4, TZmJ2, TZnB16, MYpJn8, TZnG2, and MYnE2. Inactivated bacterial cultures: inoculated with inactivated strain cultures of TZnJn3, TZnJn4, MYnB4, TZmJ2, TZnB16, MYpJn8, TZnG2, and MYnE2. CK: inoculated with sterilized LB medium.

The inoculation with isolates capable of nitrogen fixation, IAA secretion, siderophore and ACC deaminase production had different effects on the growth of roots (Figs. 5A-5E; Table S3). Both TZnB16 and its inactivated strain inhibited RL, RSA, RAD and NRT. Except for inactivated TZnG2, inoculation with the other strains could significantly promote RL. Among them, TznJn4 had the greatest capacity, which increased by 40.05%. Except for strain MYnE2 and inactivated MYnE2 and TZnG2, all strains had a significant influence on the RSA. Among them, TznJn3 exerted the strongest effect, which increased by 29.55%. After inoculation with strains TznJn4, MYnB4, and TZnB16, the RAD was lower than that of the control group, but there was no significant difference (*p*>0.05). All other treatments increased the RAD to varying degrees. Among them, MYnE2 had the most significant effect, which increased by 18.51%. Except for strain TZnB16, other treatment groups significantly increased the NRT by 0.5-33.00%. Among them, strain TznJn4 exerted the strongest effect. After inoculation with strain MYnB4, inactivated MYnB4, TZmJ2, and MYnE2, the RDW was lower than that of the control group, but there was no significant difference (*p* > 0.05). Other treatment groups all increased RDW by 2.29-58.85%. Among them, TZnG2 had the strongest effect, increasing by 58.85%.

After inoculation with TZnB16, MYnE2 and inactivated MYpJn8, the SH was lower than that of the control group, but there was no significant difference (*p* > 0.05). All the other strains had a positive effect on SH, among which TznJn3 exerted the strongest effect, which increased by 30.76% (Figs. 5F-5H; Table S4). After inoculation with inactivated MYnB4, the SD was slightly smaller than that of the control group, but there was no significant difference (*p* > 0.05). Other treatment groups all increased the SD by 1.85-28.24%. Moreover, TznJn4 had the strongest effect, which increased by 28.24%. All isolates increased the SDW by 8.20-54.55%. Among them, TznJn3 had the strongest effect, which increased by 54.55%.

### Correlation Analysis between Plant Growth-Promoting Characteristics and the Growth-Promoting Effects Effect of Cold-Adapted PGPB Inoculants

**Fig. 6:**
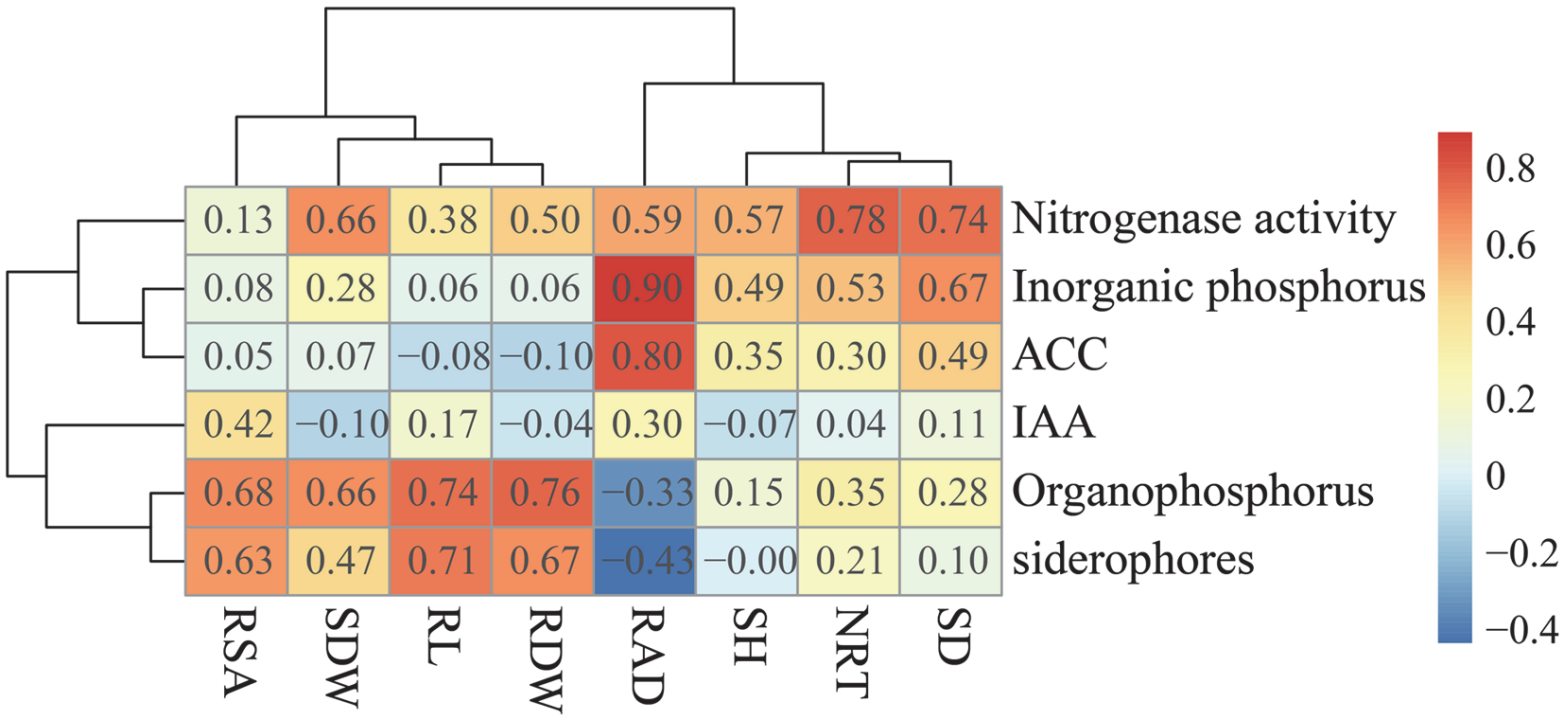
Pearson correlation analysis and heatmap. Longitudinal indexes were the plant growth-promoting characteristics, while transverse indexes were the development information of the roots and aboveground parts of *Elymus nutans*. Moreover, the corresponding value of the heatmap was the Pearson correlation coefficient r, which ranged from -1 to 1. Furthermore, r < 0 was a negative correlation, r > 0 was a positive correlation.

According to the Pearson correlation analysis, inoculation with cold-adapted PGPB influenced the growth characteristics of *E. nutans* (Fig. 6). The abilities of nitrogen fixation and inorganic phosphate solubilization were positively correlated with all plant characteristics. Furthermore, nitrogen-fixing ability exhibited an extremely significant (*p* < 0.01) and positive correlation with NRT (r = 0.778), SD (r = 0.744) and SDW (r = 0.657). Inorganic phosphate solubilization ability was extremely significantly (*p* < 0.01) and positively correlated with RAD (r = 0.896) and SD (r = 0.668). Except for a weak negative correlation with RL and RDW (*p* > 0.05), ACC deaminase activity was positively correlated with other growth characteristics, especially with RAD (r = 0.803, *p* < 0.01). Except for a weak negative correlation with RAD (*p* > 0.05), organic phosphate solubilization ability exhibited a positive correlation with other growth characteristics, especially RDW (r = 0.764, *p* = 0.001), RL (r = 0.74, *p* = 0.002), RSA (r = 0.677, *p* = 0.006) and SDW (r = 0.662, *p* = 0.007). Siderophore production ability was significantly positively correlated with RL, RDW, and RSA (*p* < 0.05) but weakly negatively correlated with SH (*p* > 0.05) and RAD (*p* > 0.05). In general, the nitrogen fixation and phosphorus solubility of cold-adapted PGPB significantly promoted the development of the roots and aboveground parts of *E. nutans*. However, ACC deaminase and siderophore production capacity mainly had significant effects on roots development.

## Discussion

PGPB with different adaptability and functions could be isolated from different habitats and sample sources. They were identified from the rhizosphere of pioneer plants growing on mine tailings [34], wheat [35], sunflower [36], forage grass [37], halophytes [10, 38], forest trees [12], and plateau plants[21] . The results showed that PGPB varied with the geographical environment and plant species. A total of 67 cold-adapted PGPB in our study belonged to eight genera, of which *Pseudomonas* and *Serratia* were the dominant genera. These results indicated that the PGPB were sensitive to external factors, such as soil fertility, soil physical properties, cultivation measures, and crop varieties. Different plant species have specific PGPB. Therefore, it is necessary to carry out targeted strain screening based on differences in the geographical environment and plant species to develop specific microbial inoculants.

At present, the main method to screen cultivable microorganisms is to use different selected media by the plate dilution coating method. However, culture medium, culture conditions, and the proficiency of operators affect the results [39]. For example, some low-abundance bacterial strains tend to be lost during serial dilution. In our study, three selected media were used to isolate PGPB. The number of rhizosphere bacteria was 1.94 times that of endophytic bacteria, indicating that PGPB were mainly distributed in the rhizosphere, which was consistent with previous reports [10, 34, 39, 40]. The rhizosphere is the most active region of plant-soil-microorganism interactions. Plants employ beneficial soil microorganisms to colonize the rhizosphere through root exudates, promoting their own growth and development [17]. In turn, these microorganisms mineralize organic substances by secreting various extracellular enzymes and secondary metabolites, thus increasing the content of available nutrients in the soil, and promoting the uptake of nutrients by plants [13, 41]. However, another possibility is that the relative abundance of endophytic bacteria is low, leading to their loss during continuous dilution. Therefore, more methods should be used to compensate for the deficiency of a single method in screening strain resources.

Based on the desire for environmentally friendly fertilizer, the exploitation of PGPB with new functions or multiple functions has attracted the attention of researchers [16, 40, 42]. In our study, cold-adapted PGPB was multifunctional, and *Pseudomonas* was the dominant genus in the rhizosphere of the four assayed plant species. *Pseudomonas* spp. are important PGPB used as biofertilizers, and can enhance crop yield by direct and indirect mechanisms [9, 19]. Previous studies have confirmed that *Pseudomonas* can be used as a cold-adapted PGPB microbial inoculant, especially for agricultural production in regions with a low climate and short growing season [43, 44]. More than 94.03% of the cold-adapted PGPB had more than two growth-promoting characteristics in our study. Compared with single growth-promoting functional inoculants, multifunctional PGPB have higher environmental adaptability, rhizosphere colonization ability, and broad-spectrum efficacy, which makes them more suitable as multifunctional microbial fertilizers in cold areas [9, 19, 45].

The PGPB with different characterization have different growth-promoting effects. In our study we found that inoculation with the twelve cold-adapted PGPB strains possessing different characteristics had different influences on the growth of the roots and aboveground parts of *E. nutans*. The Pearson correlation coefficient showed that nitrogen fixation ability had the greatest impact on NRT and SD, while the inorganic phosphorus-solubilizing ability made the greatest contribution to RAD. A PGPB strain CKMV1 isolated from the rhizosphere of *Valeriana jatamansi* exhibited similar functions as our isolates [46]. Combined inoculation with PGPB capable of fixing nitrogen and dissolving phosphorus without antagonism showed a significant growth-promoting effect on oat (*A. sativa*), alfalfa (*Medicago sativa*), cucumber (*Cucumis sativus*) [9] and maize plants [46]. The above results indicated that the nitrogen fixation and phosphorus solubilization capacities of PGPB might be involved in the mechanism underlying their ability to promote plant growth. Other studies have shown that the development of the roots and the increase in aboveground biomass are mainly related to the secretion of IAA by PGPB [47]. The inoculation of rapeseed seeds with *Pseudomonas putida* GR12-2, which produces low IAA levels, resulted in a 20-30% increase in primary root elongation. Furthermore, another variant of the strain that produces higher levels of IAA promoted the development of many secondary roots [48]. Our research showed that the two strains TZnB16 and TZmJ2, which exhibited the strongest IAA secretion capacity, showed no positive effect on the root growth of *E. nutans*, even though inoculation with TZnB16 resulted in weak inhibitory effects on RL, RSA, RAD and NRT. However, they had positive effects on SH, SD, SDW and RDW. We speculate that this is likely due to the superposition of multiple growth-promoting functions of these strains, but the corresponding mechanism needs to be further studied.

ACC deaminase has been proved to be an important mechanism by which PGPB promotes plant growth and improves resistance to abiotic stress [18, 49]. Inoculation with rhizosphere bacteria possessing ACC deaminase activity had positive effects on both the development of roots and the aboveground parts of plants [50]. In our study, the inoculation of *E. nutans* with the two strains TZnG2 and MYpJn8, which had the highest ACC deaminase activity, resulted in similar results. Pearson correlation analysis (Fig. 6) showed that ACC deaminase activity had the most significant effect on the RAD. Organophosphorus-degrading bacteria decompose insoluble organophosphorus of soil into soluble small molecules by secreting organophosphorus enzymes, such as phosphatase, phytase and nuclease, thus providing more phosphorus sources for plants and promoting their development [51]. Organophosphorus-degrading bacterial strains MYmG3 and MYmG4 significantly increased the RL and RSA of *E. nutans* and promoted SDW and RDW. Siderophores are essential for iron nutrition in microorganisms and are an important mechanism for the inhibition of soil-borne diseases [44]. The thirty-five cold-adapted PGPB strains isolated in our study showed inhibitory abilities against four plant pathogens, indicating that they can play an indirect role in biological control for plants. Most of them belong to *Pseudomonas*, consistent with previous descriptions [52, 53].

Our study showed that the ability of PGPB to dissolve phosphorus was negatively correlated with the medium pH. The lower the pH value, the greater the phosphate solubility, but there was no linear relationship (Fig. 1). It is widely believed that functional bacteria mainly dissolve insoluble phosphorus by secreting organic acids, protons and polysaccharides [54]. It can be speculated that phosphate-dissolving strains isolated in our study (mainly *Pseudomonas*) mostly depended on the secretion of organic acids and protons to dissolve tricalcium phosphate in the medium. Some genera, such as *Pseudomonas* sp., *Pantoea* sp., *Mycobacterium* sp., *Mycoplasma* sp., and *Acinetobacter* sp. have exhibited phosphate solubilization ability at low temperatures from 4 to 16°C [55]. Some genera such as *Pseudomonas* sp., *Pantoea* sp., *Mycobacterium* sp., *Mycoplasma* sp., and *Acinetobacter* sp. have exhibited phosphate solubilization ability at low temperatures from 4 to 16°C [56]. Crop seeding usually occurs in early spring or autumn when the soil temperature is low. The cold-adapted PGPB isolated from the alpine grasslands of the Qilian Mountains can be developed as seed dressing agents, which can play a role in the disease resistance and yield preservation of crops. Furthermore, due to the influence of high altitude and low temperature, vegetation restoration after degradation of the Qinghai-Tibet Plateau is relatively slow. Our cold-adapted PGPB has the characteristics of promoting plant growth and inhibiting pathogenic fungi, as well as adapting to the plateau ecological environment, leading to their application potential in plateau vegetation restoration and as a resource for research on biological fertilizers and pesticides.

## Supplemental Materials

Supplementary data for this paper are available on-line only at http://jmb.or.kr.

## Figures and Tables

**Table 1:** Basic information of the sampling sites.

Sampling site	Longitude and latitude	Altitude (m)	Soil types	Annual average temperature (°C)	Annual precipitation (mm)	Climate types	Main vegetation
Site TZ	37°11'N, 102°48'E	2940	Subalpine meadow soil and chernozem	-0.1~0.6	370~468	A continental cold, wet and semiarid climate	*Kobresia Bellardii*, *Carex* spp., *Elymus nutans*, *Poa pratensis*, *Oxytropis ochrocephala,* etc.
Site MY	37°37'N, 109°11'E	3240	Alpine meadow soil	-2.5~-0.4	420~860	A plateau continental climate	*Kobresia Bellardii, Stipa aliena, Elymus Nutans, Poa pratensis, Potentilla fruticosa,* etc.

**Table 2:** Isolation of nitrogen-fixing and phosphate-dissolving strains.

Sampling site	Plant	Sample ID	NFM agar plates		NBRIP agar plates		Mongina agar plates	

			Rhizosphere	root	Rhizosphere	root	Rhizosphere	root
Tianzhu	*Bromus inermis* Leyss	TZnB/TZpB/TZmB	15	12	6	0	4	1
Menyuan	*Bromus inermis* Leyss	MYnB/MYpB/MYmB	10	4	11	2	5	2
Tianzhu	*Elymus nutans*	TZnE/TZpE/TZmE	6	7	5	0	5	2
Menyuan	*Elymus nutans*	MYnE/MYpE/MYmE	4	8	8	5	4	2
Tianzhu	*Avena sativa* L.	TZnG/TZpG/TZmG	12	15	5	0	4	1
Menyuan	*Avena sativa* L.	MYnG/MYpG/MYmG	15	6	12	2	9	2
Tianzhu	*Poa annua* L.	TZnJ/TZpJ/TZmJ	17	14	4	0	8	1
Menyuan	*Poa annua* L.	MYnJ/MYpJ/MYmJ	6	6	7	4	6	5

In the bacterial strain number, the first two letters "TZ" and "MY" represent Tianzhu and Menyuan, respectively. The third letters "n", "p" and "m" represent NFM medium, NBRIP medium and Mongina medium, respectively. The fourth letter represents the corresponding host plants. The fifth letter represents sample number, and the letter "n" stands for endophytic bacteria (if any).

**Table 3:** Plant growth-promoting characteristics of 67 cold-adapted PGPB strains.

Strains	Organophosphorus solubility(μg/ml^-1^)	Inorganic phosphorus solubility(μg/ml^-1^)	IAA contents(μg/ml^-1^)	Nitrogenase activity (nmol/mg•protein•h)	siderophores production capacity(μg/ml^-1^)	ACC deaminase activity [CH_3_CH_2_COCOOH μmol/mg•protein•h]	Inhibition of pathogenic fungi			

							*Fusarium oxysporum*	*Rhizoctonia solani*	*Helminthosporium tritici-vulgaris*	*Alternaria solani*
TZnB8	3.21±0.16	63.26±4.50	–	276±15.24	0.645±0.008	–	35.33±1.15	52.37±1.48	27.56±0.77	24.07±1.89
TZnB11	15.21±0.34	86.56±2.61	24.95±1.32	181.94±12.40	0.642±0.002	–	44.67±1.15	55.11±1.54	41.33±1.15	52.37±1.48
TZnB13	4.22±0.28	31.53±2.82	–	181.94±9.18	0.381±0.052	–	33.33±2.31	23.41±1.03	–	45.48±0.90
TZnB16	10.27±0.15	41.18±1.01	48.95±3.93	185.16±11.37	0.627±0.03	–	34.00±2.00	17.19±1.41	–	38.59±1.22
TZnBn1	7.60±0.49	51.73±3.04	–	336.32±20.98	–	–	–	–	–	–
TZnBn2	6.28±0.39	88.02±3.04	–	358.12±16.19	–	–	–	–	–	–
TZnBn3	7.08±0.51	101.12±2.89	9.45±1.03	346.33±15.55	–	–	–	–	–	–
TZnBn6	5.21±0.46	–	44.85±3.03	375.27±15.07	0.657±0.044	–	28.67±1.15	24.41±1.96	28.22±1.68	34.44±1.39
TZnBn7	8.14±0.62	–	–	194.81±11.38	0.464±0.05	–	35.33±1.15	23.41±1.03	–	45.48±0.90
TZnBn10	2.82±0.31	130.40±7.05	–	349.18±4.92	–	–	–	–	–	–
TZnBn11	3.21±0.24	127.10±4.10	28.15±2.21	447.46±20.65	0.593±0.021	–	35.33±1.15	43.41±1.03	–	45.48±0.90
TZnBn12	4.34±0.67	–	8.57±0.52	196.95±15.28	0.593±0.03	–	46.67±1.15	51.70±0.51	–	51.70±0.51
TZnE1	–	–	–	280.87±11.89	0.328±0.024	–	–	–	–	–
TZnE12	–	56.27±1.64	30.71±1.13	449.96±18.08	0.464±0.049	–	43.33±1.15	44.81±1.05	–	45.48±0.90
TZnE14	8.33±0.83	91.65±6.17	28.15±0.57	505.70±40.79	0.295±0.029	–	54.00±2.00	56.52±1.30	–	53.44±0.51
TZnEn3	8.67±1.14	–	–	362.05±18.31	–	–	–	–	–	–
TZnEn4	6.21±0.51	122.44±6.17	–	330.60±21.28	–	–	–	–	–	–
TZnG2	–	–	17.13±1.62	235.55±21.36	–	3.42±0.31	–	–	–	–
TZnG7	–	–	–	223.75±12.62	0.427±0.016	–	–	–	41.33±1.15	–
TZnG12	–	54.87±4.60	8.57±0.97	545.23±26.61	–	–	–	–	–	–
TZnG17	–	156.3±5.04	35.37±2.25	241.45±13.11	0.384±0.032	–	–	–	41.33±1.15	–
TZnG21	–	–	–	564.27±7.72	–	–	–	–	–	–
TZnGn1	3.83±0.63	36.53±1.29	9.45±0.65	235.55±16.52	–	–	–	–	–	–
TZnGn4	6.18±0.59	73.18±2.36	–	179.80±7.54	0.498±0.019	–	50.67±2.31	52.37±1.48	25.11±1.54	52.44±0.77
TZnGn5	–	172.44±9.25	–	174.44±15.64	0.434±0.032	–	35.33±1.15	30.30±1.58	–	43.48±1.30
TZnGn8	9.28±0.83	192.81±4.77	–	187.30±10.03	–	–	–	–	–	–
TZnGn11	1.81±0.10	126.10±7.33	–	283.79±9.10	–	–	–	–	–	–
TZnGn13	12.28±0.88	134.69±6.97	3.24±0.76	193.74±14.54	–	–	–	–	–	–
TZnGn14	32.53±4.62	257.12±35.26	4.73±0.18	217.17±9.40	–	–	–	–	–	–
TZnGn15	–	–	–	169.08±11.93	–	–	–	–	–	–
TZnJ8	7.87±1.44	108.03±9.92	23.17±1.13	214.11±16.40	0.391±0.017	–	49.33±2.31	53.78±0.39	6.22±0.39	51.70±0.51
TZnJ9	–	29.84±7.78	–	329.17±10.77	–	–	–	–	–	–
TZnJ10	–	–	36.19±1.76	434.73±25.91	0.638±0.02	–	–	–	25.11±1.54	–
TZnJ14	–	76.47±6.94	7.27±0.29	543.58±21.85	–	–	–	–	–	–
TZnJ15	–	–	–	413.36±23.67	–	–	–	–	–	–
TZnJn1	10.76±2.51	–	3.26±0.47	535.72±15.68	–	–	–	–	–	–
TZnJn3	–	–	13.67±1.20	867.29±60.07	–	–	–	–	–	–
TZnJn4	–	92.75±4.65	14.95±1.00	907.31±43.92	–	–	–	–	–	–
TZnJn7	–	–	–	210.89±7.77	–	–	–	–	–	–
TZnJn8	26.78±1.88	180.43±8.80	–	210.89±13.03	0.402±0.005	–	22.67±2.31	23.41±1.03	27.56±0.77	33.78±0.39
TZnJn10	5.42±0.41	88.16±8.54	3.76±0.14	184.09±8.13	–	–	–	–	–	–
TZnJn11	23.37±1.37	172.14±19.13	–	189.45±9.34	–	–	–	–	–	–
MYnB4	–	–	–	180.87±6.17	0.037±0.017	–	51.67±4.04	56.52±1.30	54.44±1.39	53.78±0.39
MYnB7	21.72±2.93	–	26.89±1.33	210.89±18.63	0.427±0.026	–	38.00±2.00	46.15±1.78	–	45.48±0.90
MYnB15	12.65±2.88	136.09±4.57	–	265.56±8.02	0.428±0.034	–	55.33±1.15	53.11±1.02	–	56.52±1.30
MYnE1	7.75±1.68	93.85±0.48	–	335.35±14.01	0.708±0.071	–	41.33±1.15	40.59±2.44	41.33±1.15	43.48±1.30
MYnE2	–	–	6.24±0.21	306.73±20.46	0.057±0.011	–	46.00±2.00	52.04±1.70	31.67±1.53	52.04±1.70
MYnEn5	8.97±1.08	53.01±1.76	–	174.44±10.39	0.558±0.037	–	38.67±1.15	50.30±1.22	6.22±0.39	45.48±0.90
MYnG2	17.63±0.43	166.95±14.31	16.03±0.39	443.31±16.77	–	–	–	–	–	–
MYnG8	12.23±1.90	205.46±9.29	–	341.29±7.60	–	–	–	–	–	–
MYnG15	–	–	5.42±0.16	305.16±4.62	–	–	–	–	–	–
TZpB3	9.63±1.72	163.26±13.67	–	246.32±12.79	0.595±0.041	–	11.33±1.15	–	15.56±0.96	–
TZpB4	7.34±0.36	186.56±14.77	–	184.08±13.71	0.558±0.046	–	28.67±1.15	27.56±0.77	–	–
TZpE5	10.32±1.73	201.80±7.66	6.72±0.40	80.86±2.61	0.25±0.019	–	22.00±2.00	–	–	–
TZpG2	15.63±1.49	194.62±11.86	–	135.54±12.83	0.06±0.016	–	34.00±2.00	52.37±1.48	51.00±1.00	52.78±0.69
TZmJ2	28.67±1.56	–	46.44±3.50	180.86±9.44	0.591±0.022	–	41.33±1.15	33.78±0.39	–	–
TZmJ8	25.07±1.24	173.41±12.76	–	214.10±16.41	0.606±0.028	–	46.67±2.31	53.78±0.39	6.22±0.39	52.37±1.48
TZpJ3	8.27±0.47	233.37±19.39	7.27±0.72	–	–	–	–	–	–	–
MYpB7	–	387.6±33.90	17.29±1.05	194.08±11.7	–	1.21±0.30	–	–	–	–
MYmE1	27.84±0.59	182.14±15.69	–	–	0.739±0.013	–	33.33±1.15	39.93±2.11	48.22±1.68	39.93±2.11
MYpEn7	–	310.91±22.50	–	174.43±4.64	–	1.23±0.18	–	–	–	–
MYmG1	26.67±2.55	161.12±8.36	15.39±0.16	135.42±6.99	0.377±0.023	–	5.33±1.15	37.26±1.10	32.00±2.00	–
MYmG3	43.89±4.39	–	12.75±0.57	–	0.35±0.013	–	–	52.37±1.48	48.56±2.22	–
MYmG4	38.66±2.44	115.25±6.43	–	279.79±8.39	0.337±0.033	–	29.33±2.31	–	24.44±1.39	–
MYmG9	29.73±0.90	173.31±9.14	–	187.306±5.75	0.614±0.017	–	35.33±1.15	35.11±1.54	31.33±1.15	23.41±1.03
MYpJn1	18.53±1.14	414.43±26.96	–	335.71±9.49	–	1.23±0.15	–	–	–	–
MYpJn8	–	460.19±31.34	26.84±1.29	110.85±7.62	–	2.37±0.27	–	–	–	–
