# Supplementary material for: Isolation and Characterization of Cold-Adapted PGPB and Their Effect on Plant Growth Promotion
Source: J Microbiol Biotechnol. 2021 Jul 15;31(9):1218–30. doi: 10.4014/jmb.2105.05012 (PMC9705895; doi:10.4014/jmb.2105.05012)
Supplement: Supplementary file 1 [file jmb-31-9-1218-supple.pdf]

J. Microbiol. Biotechnol.
https://doi.org/10.4014/jmb.2105.05012

Table S1. Effect of phosphate-dissolving strains on the development of root systems

Treatment	Root length (RL)		Root surface area (RSA)		Root average diameter (RAD)		Number of root tips (NRT)		Root dry weight (RDW)	
	Value/cm	Rate of increase /%	Value/cm^2	Rate of increase /%	Value/mm	Rate of increase /%	count	Rate of increase /%	Value/g	Rate of increase /%
MYmG3	78.29±5.12ab	58.93	10.94±1.22a	77.89	0.41±0.07c	-3.28	155.00±19.67b	30.25	0.019±0.002ab	58.33
inactivated MYmG3	53.49±6.24c	8.59	6.92±0.92c	12.52	0.41±0.07c	-2.97	116.00±12.12c	-2.52	0.017±0.002bc	41.67
MYmG4	83.48±4.42a	69.47	9.75±0.106ab	58.54	0.54±0.07b	25.76	198.67±13.87a	66.95	0.021±0.002a	75.00
inactivated MYmG4	81.10±11.64ab	64.63	9.54±0.32ab	55.12	0.39±0.04c	-9.60	183.00±16.09ab	53.78	0.018±0.003abc	50.00
MYpJn1	68.27±6.01b	38.59	9.07±0.73b	47.40	0.65±0.02a	51.29	193.00±18.00a	62.18	0.017±0.001abc	41.67
inactivated MYpJn1	54.09±7.27c	9.80	8.59±0.54b	39.67	0.42±0.06c	-1.41	188.33±13.01a	58.26	0.014±0.001cd	16.67
MYpJn8	69.68±10.92b	41.46	9.49±1.62ab	54.31	0.67±0.04a	57.38	176.67±16.17ab	48.46	0.016±0.002bc	33.33
inactivated MYpJn8	53.24±3.06c	8.08	7.06±0.61c	14.80	0.34±0.04c	-20.84	174.67±12.01ab	46.78	0.015±0.001cd	25.00
CK	49.26±2.86c	/	6.15±0.42c	/	0.43±0.07c	/	119.00±22.72c	/	0.012±0.001d	/

Different letters represent significant statistical differences at $P<0.05$. The same below.

Table S2. Effect of phosphate-dissolving strains on aboveground biomass

Treatment	Stem height (SH)		Stem diameter (SD)		Stem dry weight (SDW)	
	Value/cm	Rate of increase /%	Value/mm	Rate of increase /%	Value/g	Rate of increase /%
MYmG3	41.37±2.10ab	5.00	1.33±0.10bcd	20.91	0.071±0.004ab	51.06
inactivated MYmG3	39.00±1.32b	-1.02	1.25±0.06de	13.64	0.053±0.005c	12.77
MYmG4	43.20±2.98ab	9.64	1.47±0.06ab	33.64	0.077±0.004a	63.83
inactivated MYmG4	39.57±1.72b	0.43	1.23±0.06de	11.82	0.067±0.004ab	42.55
MYpJn1	45.33±1.04a	15.05	1.55±0.07a	40.91	0.076±0.005a	61.70
inactivated MYpJn1	39.83±1.53b	1.09	1.26±0.09de	14.55	0.050±0.005c	6.38
MYpJn8	42.70±2.71ab	8.38	1.45±0.11abc	31.82	0.062±0.007b	31.91
inactivated MYpJn8	39.77±3.61b	0.94	1.30±0.11cd	18.18	0.049±0.005c	4.26
CK	39.40±3.92b	/	1.10±0.08e	/	0.047±0.006c	/

Different letters represent significant statistical differences at $P<0.05$.

Table S3. Effect of nitrogen-fixing, IAA, siderophore and ACC deaminase producing strains on the development of root system

Treatment	Root length (RL)		root surface area (RSA)		root average diameter (RAD)		Number of root tips (NRT)		Root dry weight (RDW)	
	Value/cm	Rate of increase /%	Value/cm^2	Rate of increase /%	Value/mm	Rate of increase /%	count	Rate of increase /%	Value/g	Rate of increase /%
TznJn3	197.55±13.10b	21.45	28.56±1.19a	29.55	0.48±0.04abc	9.09	602.33±47.61ab	28.25	0.044±0.005ab	37.5
inactivated TznJn3	182.28±19.27bc	12.06	24.77±1.97cd	12.34	0.44±0.02bcd	0	545.67±43.66c	16.18	0.038±0.004bc	18.23
TznJn4	227.81±18.27a	40.05	28.13±1.61ab	27.57	0.42±0.02cd	-4.74	624.67±38.37a	33	0.041±0.005b	27.71
inactivated TznJn4	181.63±13.77bc	11.66	24.48±2.16cde	11.02	0.44±0.02bcd	0	577.67±12.10abc	22.99	0.040±0.007b	26.46
MYnB4	177.94±7.34bc	9.39	24.39±0.56cde	10.61	0.43±0.01cd	-4.06	472.00±16.70def	0.5	0.029±0.001d	-9.27
inactivated MYnB4	169.16±4.52cd	4	23.55±0.88cde	6.8	0.42±0.08cd	-5.64	478.67±18.88de	1.92	0.029±0.001d	-8.23
TZmJ2	168.50±9.71cd	3.59	24.75±0.46cd	12.24	0.50±0.03ab	12.42	472.67±21.20def	0.64	0.041±0.005b	29.06
inactivated TZmJ2	163.72±7.13cd	0.65	22.57±2.03de	2.36	0.46±0.03abcd	3.84	468.00±24.56def	-0.36	0.030±0.002d	-6.87
TZnB16	148.81±7.54de	-8.51	21.57±1.45e	-2.18	0.43±0.01bcd	-2.71	441.67±28.02ef	-5.96	0.038±0.006bc	17.81
inactivated TZnB16	136.32±15.53e	-16.19	21.53±0.69e	-2.36	0.40±0.04d	-9.93	424.00±9.00f	-9.72	0.033±0.002cd	2.29
MYpJn8	180.17±9.56bc	10.76	25.85±1.11bc	17.23	0.47±0.04abc	6.77	559.00±30.51bc	19.02	0.050±0.002a	55.52
inactivated MYpJn8	173.25±10.25c	6.51	22.68±1.61de	2.86	0.44±0.04bcd	0	486.33±10.07de	3.55	0.049±0.005a	54.58
TZnG2	170.31±15.80cd	4.7	23.90±2.02cde	8.39	0.48±0.00abc	8.35	496.00±32.05d	5.61	0.051±0.002a	58.85
inactivated TZnG2	161.51±9.31cd	-0.71	21.56±2.20e	-4.26	0.46±0.03bcd	2.93	480.33±6.66de	2.27	0.041±0.001b	27.4
MYnE2	167.47±12.52cd	2.96	21.89±1.19de	-0.73	0.53±0.04a	18.51	493.33±28.50de	5.04	0.050±0.002a	56.67
inactivated MYnE2	162.78±7.08cd	0.07	21.69±1.67e	-1.63	0.50±0.04ab	12.64	476.33±31.53de	1.42	0.032±0.003cd	-1.35
CK	162.66±8.11cd	/	22.05±0.91de	/	0.44±0.01bcd	/	469.67±21.83def	/	0.032±0.006cd	/

Different letters represent significant statistical differences at $P<0.05$.

Table S4. Effect of nitrogen-fixing, IAA, siderophore and ACC deaminase producing strains on the development of aboveground parts

Treatment	Stem height (SH)		Stem diameter (SD)		Stem dry weight (SDW)	
	Value/cm	Rate of increase /%	Value/mm	Rate of increase /%	Value/g	Rate of increase /%
TznJn3	45.33±3.51a	30.76	1.66±0.12bc	15.51	0.195±0.007a	54.55
inactivated TznJn3	42.33±2.08ab	22.1	1.64±0.09bc	14.12	0.184±0.013abc	45.93
TznJn4	43.33±3.79a	24.99	1.85±0.11a	28.24	0.189±0.012ab	50.21
inactivated TznJn4	40.67±3.21abc	17.3	1.61±0.07bcd	12.04	0.177±0.008bc	40.79
MYnB4	44.00±1.73a	26.91	1.55±0.08cde	7.41	0.151±0.010d	20.13
inactivated MYnB4	45.00±3.00a	29.8	1.43±0.08e	-0.46	0.136±0.013e	8.2
TZmJ2	45.00±5.29a	29.8	1.61±0.10bcd	11.81	0.155±0.010d	22.7
inactivated TZmJ2	37.00±3.46bcd	6.72	1.52±0.06cde	5.79	0.150±0.003d	19.13
TZnB16	31.33±2.08d	-9.62	1.71±0.03b	18.52	0.172±0.001c	36.19
inactivated TZnB16	36.00±4.00cd	3.84	1.53±0.05cde	6.25	0.158±0.005d	25.48
MYpJn8	37.00±2.00bcd	6.72	1.65±0.08bc	14.35	0.190±0.008ab	50.53
inactivated MYpJn8	34.00±3.46d	-1.93	1.54±0.07cde	7.18	0.177±0.007bc	40.37
TZnG2	35.67±3.06cd	2.87	1.71±0.04b	18.52	0.187±0.006ab	48.23
inactivated TZnG2	35.33±2.31cd	1.91	1.64±0.05bc	13.89	0.186±0.003ab	47.91
MYnE2	34.00±2.00d	-1.93	1.76±0.04ab	21.99	0.189±0.007ab	49.84
inactivated MYnE2	42.33±3.21ab	22.1	1.47±0.08de	1.85	0.157±0.007d	24.42
CK	34.67±2.08d	/	1.44±0.11e	/	0.126±0.002e	/

Different letters represent significant statistical differences at $P<0.05$.
